# A Novel Hybrid Deep Learning Method for Predicting the Flow Fields of Biomimetic Flapping Wings

**DOI:** 10.3390/biomimetics9020072

**Published:** 2024-01-25

**Authors:** Fujia Hu, Weebeng Tay, Yilun Zhou, Boocheong Khoo

**Affiliations:** 1 Department of Energy and Power Engineering, North University of China, Taiyuan 030051, China; hfj_715@163.com; 2 Department of Fluid Machinery and Engineering, Xi’an Jiaotong University, Xi’an 710049, China; zhouyilun1998@163.com; 3 Temasek Laboratory, National University of Singapore, 5A, Engineering Drive 1, #02-02, Singapore 117411, Singapore; 4 Department of Mechanical Engineering, National University of Singapore, 10 Kent Ridge Crescent, Singapore 119260, Singapore; mpekbc@nus.edu.sg

**Keywords:** bio-inspired flapping wings, physics-informed neural network, data-driven, deep learning, computational fluid dynamics

## Abstract

The physics governing the fluid dynamics of bio-inspired flapping wings is effectively characterized by partial differential equations (PDEs). Nevertheless, the process of discretizing these equations at spatiotemporal scales is notably time consuming and resource intensive. Traditional PDE-based computations are constrained in their applicability, which is mainly due to the presence of numerous shape parameters and intricate flow patterns associated with bionic flapping wings. Consequently, there is a significant demand for a rapid and accurate solution to nonlinear PDEs, to facilitate the analysis of bionic flapping structures. Deep learning, especially physics-informed deep learning (PINN), offers an alternative due to its great nonlinear curve-fitting capability. In the present work, a hybrid coarse-data-driven physics-informed neural network model (HCDD-PINN) is proposed to improve the accuracy and reliability of predicting the time evolution of nonlinear PDEs solutions, by using an order-of-magnitude-coarser grid than traditional computational fluid dynamics (CFDs) require as internal training data. The architecture is devised to enforce the initial and boundary conditions, and incorporate the governing equations and the low-resolution spatiotemporal internal data into the loss function of the neural network, to drive the training. Compared to the original PINN with no internal data, the training and predicting dynamics of HCDD-PINN with different resolutions of coarse internal data are analyzed on the problem relevant to the two-dimensional unsteady flapping wing, which involves unsteady flow features and moving boundaries. Additionally, a hyper-parametrical study is conducted to obtain an optimal model for the problem under consideration, which is then utilized for investigating the effects of the snapshot and fraction of the coarse internal data on the HCDD-PINN’s performances. The results show that the proposed framework has a sufficient stability and accuracy for solving the considered biomimetic flapping-wing problem, and its great potential means that it can be considered as an alternative to accelerate or replace traditional CFD solvers in the future. The interested variables of the flow field at any instant can be rapidly obtained by the trained HCDD-PINN model, which is superior to the traditional CFD method that usually needs to be re-run. For the three-dimensional and optimization problems of flapping wings, the advantages of the proposed method are supposedly even more apparent.

## 1. Introduction

Deep learning (DL) has gained great attention over the last decade owing to its enormous breakthroughs in many fields, such as image processing [1], speech recognition, and disease diagnosis [2]. Recently, because of its excellent abilities in handling strong nonlinearity and high dimensionality, DL has been widely tested in solving fluid dynamics and for envisaging, accelerating, or replacing computational fluid dynamic (CFD) simulations in the future without compromising accuracy. Ling et al. [3] achieved the first combination of a deep neural network (DNN) and fluid mechanics by constructing the deep-learning RANS turbulence model. Convolution neural networks (CNNs) can accurately learn the characteristics of flow around a cylinder [4,5], and can be combined with Multilayer Perceptron (MLP) to efficiently and accurately learn the incompressible laminar steady flow fields over airfoils [6], while combining them with long short-term memory (LSTM) allows them to learn the spatial–temporal features of turbulence dynamics [7]. However, these DL experiments heavily depend on a large amount of training data to ensure learning accuracy, and they may fail to operate when the data become sparse. Hence, it can be considered as a black box (purely data-driven and lacking physical interpretation).

Differently to the purely data-driven deep learning method, the application of a neural network for simulating the fluid physics governed by the partial differential equations (PDEs) was first tested by Dissanayake and Phan-Thien [8], where they incorporated the PDEs and boundary conditions into the residual form of the neural network. Van Milligen et al. [9] applied a similar method in the magnetohydrodynamic plasma equilibrium problem. Raissi et al. [10] reinvigorated the original approach with modern and accessible tools, referring to it as physics-informed neural network (PINN) for both forward and inverse problems. This has sparked considerable interest in improving and expanding the PINN approach to a wide range of physical situations, such as the vortex-induced vibrations (VIV) problem [11], multiscale problems [12], and the supersonic flows problem [13]. Automatic differentiation and the back-propagation algorithm are currently the dominant training approaches, choosing their derivatives with respect to the parameters (e.g., weights and biases) of the PINN model, which is suitable for dealing with derivatives at complex boundaries and in arbitrary domains. Rao et al. [14] constrained the loss function formulation of PINN through a mixed-variable scheme of Navier–Stokes equations to simulate the steady and transient laminar flows past a cylinder at low Reynolds numbers, which has been verified to improve the PINN’s trainability and the accuracy of the solution. Choi et al. [15] used mini-batch training and a weighted loss function to handle the memory error and divergence problems which occur when using a PINN model for training a chemical-reactor-like multi-reference frame system. Wu et al. [16] proposed a generative adversarial network framework by embedding Navier–Stokes equations into the residual, to efficiently and precisely generate the flow filed data past a cylinder. Cheng and Zhang [17] tested the PINN with residual neural network blocks for Burger’s equation and the Navier–Stokes (N-S) equations, which has been proven to exhibit a stronger predictive ability in fluid dynamic problems.

The proposal of PINN is an important breakthrough, since it transforms solving numerical problems to an unconstrained minimization problem. PINN exhibits numerous advantages, such as effectively training with small or no datasets, training in any region to satisfy the governing equation, and solving the inverse problem of finding unknown parameters that converge toward the true values. Ideally, the unique solution should be captured by PINN without any labeled data when the initial and boundary conditions are well imposed, which represents that the corresponding PDEs problem is well defined [18]. However, PINNs still encounter great challenges in achieving stable training and producing accurate predictions, especially when the underlying PDEs solutions contain high-frequencies or multi-scale features [19,20]. Besides being difficult to converge, the prediction of a PINN may hardly satisfy the ground truth even when the residual loss has been reduced to a relatively low value. The shortcomings and training deficiencies of the existing PINN model are extremely notable in the more unsteady and complex problems, which may result in larger errors for unsteady characteristics as time proceeds [11]. Raissi et al. [21] developed hidden fluid mechanics (HFMs) by using several snapshots of extracted concentration fields to quantitatively obtain the flow fields for several physical and biomedical problems. Kochkov et al. [22] introduced an end-to-end deep learning method to accelerate prediction and improve approximations for two-dimensional turbulent flows by using an order-of-magnitude-coarser grid than is traditionally required.

The physics of the flow past a flapping wing can be well-simulated by solving the corresponding PDEs, which involves unsteady features and large body motions. Extending the PINN approach to other complex fluid dynamic problems, such as the simulation of a flapping wing, is our main interest in the current work. To the best of our knowledge, this problem has not been solved using a PINN before. Inspired by micro air vehicles’ (MAVs) potential military and civilian applications [23], research on the aerodynamics of insect flapping flight have drawn significant attention, including flow visualization wind experiments of real insects in tethered and free-flying conditions [24,25] or the dynamically scaled-up models with prescribed kinematics [26,27], and the numerical simulations using the traditional CFD methods [28,29] or the immersed boundary method (IBM) [30,31]. It is widely proven that the unsteady aerodynamic mechanisms, including delayed leading-edge vortex (LEV) [32], wake capture [33], rapid pitch-up [33] and clap-and-fling [34], collectively contribute to the high-lift generation and energy harvest of flapping wings. Therefore, to reveal the underlying high-performance mechanism of a flapping wing, accurate and detailed information about aero-forces and capture and flow structures are of significant importance [35]. 

In the current work, we propose a hybrid coarse-data-driven physics-informed neural network model (HCDD-PINN), aiming to improve the PINN model to solve the CFD problem by utilizing sparse data obtained from the corresponding experimental and numerical studies. The verification case considered in the current study involves large body motions and unsteady features, like the flow past flapping wings. The variables of interest, such as the velocity and pressure fields, are predicted by the trained HCDD-PINN model, which is obtained by minimizing the loss of the neural network with the physics-constrained enforcement and the supplementary coarse internal data. One challenge of using HCDD-PINN is dealing with the presence of the flapping wing, which is solved by adding boundary collocation points with prescribed velocities and filtering the inner collocation points out of the domain. The vicinity of the flapping wing has sufficient collocation points to ensure prediction accuracy. The remainder of this study is structured as follows. Section 2 presents the details of the methodology for the proposed HCDD-PINN model. In Section 3, the training and predicting dynamics of the HCDD-PINN with different coarse internal data sources are analyzed in detail, and we discuss the results of the simulations. Subsequently, the effects of the snapshot and fraction of the coarse internal data are investigated in Section 4, based on the HCDD-PINN model with the optimal hyper-parameters, in order to test the stability of the HCDD-PINN and obtain the minimum amount of coarse data required to achieve sufficient accuracy. Finally, the main conclusions are summarized in Section 5.

## 2. Methodology

### 2.1. Governing Equations and CFD Solver Setup

The unsteady two-dimensional flow past a flapping wing is governed by the following incompressible Navier–Stokes equations:

(1) ∇·u=0

(2) $$\frac{\partial u}{\partial t}+u\cdot \nabla u=-\frac{1}{\rho }\nabla p+\frac{\mu }{\rho }\nabla^{2}u$$

where ∇ , ***u***, and *p* are the gradient operator, velocity vector, and pressure, respectively. 

**Figure 1:**
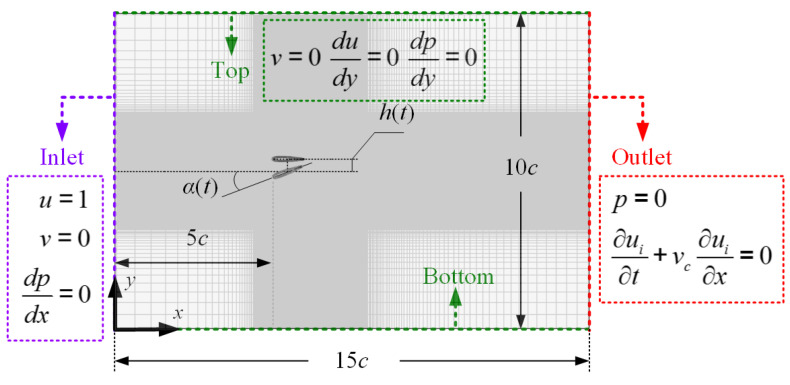
Illustration of the computational domain, boundary conditions, and kinematics of the flapping wing.

An immersed boundary method (IBM), staggered Cartesian grid, Navier–Stokes solver, which has been validated with sufficient accuracy against several problems with large body motions [30], is adopted to obtain a solution for the flapping wing problem. The wing model is a symmetrical NACA0012 airfoil, and the wing’s kinematics are prescribed by two motions: (i) translating along *y* axis (plunge) described by *h*, and (ii) rotating about the wing center (pitch) described by *α*, given by the following:

(3) $$h\left(t\right)=h_{m}\cos\left(2\pi ft\right)$$

(4) $$\alpha \left(t\right)=\alpha_{m}\sin\left(2\pi ft\right)$$

where *h_m_* and *α_m_* represent the plunge amplitude and pitch amplitude, respectively. *f* is the flapping frequency. A schematic of the wing kinematics can be found in Figure 1. The computational domain and the boundary conditions are also illustrated in the figure, which are described below.

The governing Equations (1) and (5) are solved by the IBM solver with the fractional step method, in which the presence of the solid flapping wing is represented by a force term *fc*, as illustrated by the following:

(5) $$\frac{\partial u}{\partial t}+u\cdot \nabla u=-\frac{1}{\rho }\nabla p+\frac{\mu }{\rho }\nabla^{2}u+fc.$$

All spatial derivatives are discretized using the second-order central difference scheme in a staggered grid, and the Courant–Friedrichs–Lewy (CFL) number is used for determining the timestep. The domain size is 15*c* × 10*c*, and the grid near the wing is uniform. Here, *c* is the chord length of the wing. The center of the flapping wing is placed at (0.5*c*, 0). The inlet boundary (*u* = 1, *v* = 0, and *dp*/*dx* = 0) is set 5*c* from the leading edge of the wing, while the outlet boundary ( $\partial u_{i}/\partial t+v_{c}\partial u_{i}/\partial x=0$ , and *p* = 0) is set 9*c* from the trailing edge of the wing. Here, *v_c_* is space-averaged streamwise velocity at the domain exit. The distances of the top/bottom to the wing is set to 5*c*, and the boundary condition of top/bottom is *du/dy* = 0, *v* = 0, and *dp*/*dy* = 0. More details of the IBM solver can be found in [30,36]. 

### 2.2. Hybrid Coarse Data-Driven with Physics-Informed Neural Network (HCDD-PINN) 

**Figure 2:**
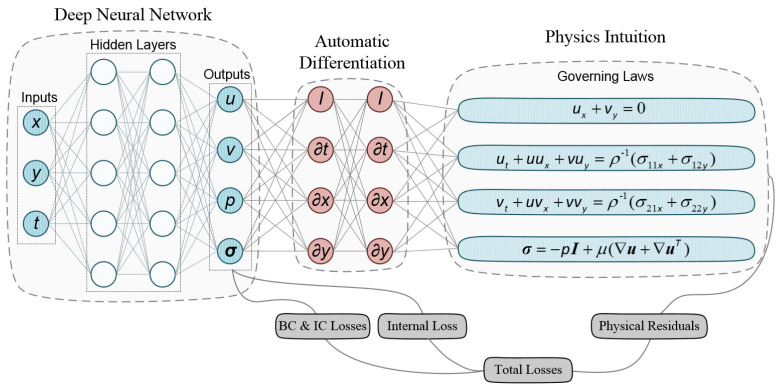
Architecture of the physics-informed neural network. For illustration purposes only, the network depicted in the figure comprises two hidden layers and five neurons per hidden layer.

The structure of the neural network consists of *N_l_* fully connected layers, which is shown in Figure 2. The input layer $X^{n}=\left(x,y,t\right)$ includes a set of independent space and time variables, while the output layer $Y^{n}=\left(u,v,p,\sigma \right)$ comprises velocity and pressure variables, and shear stress vector. The physical parameters of interest, for flow field comparisons later, consist of *u*, *v*, and *p*. All of them are evaluated because they are constrained through Navier–Stokes equations and boundary conditions simultaneously, which is different from the traditional CFD method. The hidden layers are used to solve the complex nonlinearity relations between the inputs and outputs, which construct a map ℒ to nonlinearly relate the inputs $X^{n}$ to the outputs $Y^{n}$ as:

(6) $$Y^{n}=ℒ\left(X^{n}\right)=l\left(X^{n},\theta \right).$$

Here, $l\left(\cdot ,\theta \right)\in {\mathbb{R}}^{N_{a}}\to {\mathbb{R}}^{N_{b}}$ represents the nonlinear compositional function parametrized by θ(W,b) (weight ***W***; bias ***b***), which can be expanded as below:

(7) $$l\left(\xi ;\theta \right)={ℋ}_{N_{l}}\left(\cdot ;\Theta_{N_{l}}\right)\circ {ℋ}_{N_{l}-1}\left(\cdot ;\Theta_{N_{l}-1}\right)\circ \cdot \cdot \cdot \circ {ℋ}_{1}\left(\xi ;\Theta_{1}\right)$$

where the symbol ∘ is the composition operation. The output vector at the *i*th layer (*i* = 1, 2 … *N_l_*) is calculated by the feed-forward algorithm, given by

(8) $${ℋ}_{i}\left(\xi ;\Theta_{i}\right)=h_{i}(\Theta_{i}\left[\xi^{T},1{]}^{T}\right)=h_{i}\left(\sum W_{ij}\xi_{j}+b_{i}\right)$$

where ${ℋ}_{i}\left(\cdot ;\Theta_{i}\right)\in {\mathbb{R}}^{l_{i-1}}\to {\mathbb{R}}^{l_{i}}$ and $h_{i}\left(\cdot \right)\in \mathbb{R}\to \mathbb{R}$ . Here, *l_i_* is the size of the output at the *i*th layer and $\Theta_{i}\in {\mathbb{R}}^{l_{i-1}*l_{i}+1}$ comprises the weights and biases corresponding to the layer *i*. The $\theta \equiv (\Theta_{1},\ldots ,\Theta_{i},\ldots ,\Theta_{N_{l}})$ vectors are evaluated by training the neural network. The initial weights and biases are determined by the Xavier method to accelerate the convergence of the neural network. $h_{i}\left(\cdot \right)$ denotes the activation function, and the tanh function is adopted here due to its infinitely differentiable capability.

The physics-informed neural network is constructed to calculate the fluid flow field through automatic differentiation (AD), which can be directly utilized in deep-learning-framework Tensorflow. To reduce the order of the derivative in Equation (2), the Cauchy stress tensor σ is introduced into Equation (2) as follows:

(9) $$\frac{\partial u}{\partial t}+u\cdot \nabla u=\frac{1}{\rho }\nabla \cdot \sigma$$

(10) $$\sigma =-pI+\mu \left(\nabla u+\nabla u^{T}\right)$$

which is added to the output vector of DNN. It is proved that such continuum-mechanics-based formulation benefits improve the trainability of DNN [14]. As shown in Figure 2, AD is adopted to calculate the identity operator *I* and the differential operators $\;\partial_{t},\;\;\partial_{x},\;and\;\partial_{y}$ , to obtain the partial derivatives in Equations (1), (9), and (10) for embedding the physical regularities into the loss function [37].

The loss function $\varepsilon_{loss}\;$ consists of two terms: (i) the physics-constrained term, which includes the governing partial differential equations’ loss $\varepsilon_{g}$ (Equations (1), (9), and (10)) and the boundary condition loss $\varepsilon_{bc}$ ; and (ii) the data correction term, representing the mean squared errors between the exact and predicted values, consists of the initial condition loss $\;\varepsilon_{I}$ and the coarse internal loss $\;\varepsilon_{int}$ , which are derived as follows:

(11) $$\varepsilon_{loss}=\varepsilon_{g}+\lambda_{bc/I}(\sum \varepsilon_{bc,j}+\varepsilon_{I})+\lambda_{int}\varepsilon_{int}$$

(12) $$\varepsilon_{g}=\frac{1}{N_{g}}\sum_{i=1}^{N_{g}}y^{*}{\left(x_{g}^{i}\right)}^{2}$$

(13) $$\varepsilon_{bc,j}=\frac{1}{N_{bc,j}}\sum_{i=1}^{N_{bc,j}}{\left(y_{bc,j}^{*}\left(x_{bc}^{i}\right)-y_{bc,j}\right)}^{2}$$

(14) $$\varepsilon_{I}=\frac{1}{N_{I}}\sum_{i=1}^{N_{I}}{\left(y^{*}\left(x_{I}^{i}\right)-y_{I}\right)}^{2}$$

(15) $$\varepsilon_{int}=\frac{1}{N_{int}}\sum_{i=1}^{N_{int}}{\left(y^{*}\left(x_{int}^{i}\right)-y_{int}\right)}^{2}.$$

Here, $\;\lambda_{bc/I}$ and $\lambda_{int}$ are adopted as the weighting coefficients for the initial and boundary conditions losses and the coarse internal data loss, respectively, in order to balance the weights of each part of the total loss. $y_{bc,j}^{*}$ is the boundary condition function, in which * denotes DNN’s predicted values. *N*_(.)_ denotes the number of collocation points (subscripts *g* for governing equation, *I* for initial condition, *bc* for boundary condition, and *int* for coarse internal training data). ${\left(x_{\left(.\right)}^{i}\right)}_{i=1}^{N_{\left(.\right)}}$ and $y_{\left(.\right)}$ are the input and output variables for all the collocation points of the physics-informed neural network, respectively. 

The total loss, as derived in Equation (11), is minimized by the ADAM and L-BFGS-B optimizers [38], which can constantly adjust the learning rates and possess good convergence speed. Theoretically, the solution for the N-S equations can be achieved, when the loss function is reduced to zero.

For the flapping wing problem considered in the current work, the domain and boundary setups utilized in the PINN are the same as in IBM simulation, as depicted in Figure 1. The presence of the flapping wing is achieved by some specific collocation points: (i) points at the moving boundary are added with prescribed velocities ( ${v_{x}}^{i},\;{v_{y}}^{i}$ ), as described in Equations (16) and (17); and (ii) points located within the flapping wing are all filtered out of the fluid computational space. That is,

(16) $${v_{x}}^{i}=2\pi f\alpha_{m}\cos\left(2\pi ft\right)\cdot r^{i}{n_{x}}^{i}$$

(17) $${v_{y}}^{i}=2\pi f\alpha_{m}\cos\left(2\pi ft\right)\cdot r^{i}{n_{y}}^{i}-2\pi fh_{m}\sin\left(2\pi ft\right)$$

where $r^{i}$ represents the length between the rotation center and the *i*th point at the moving boundary. ( ${n_{x}}^{i},\;{n_{y}}^{i}$ ) is the outward normal on the flapping wing the *i*th point.

**Figure 3:**
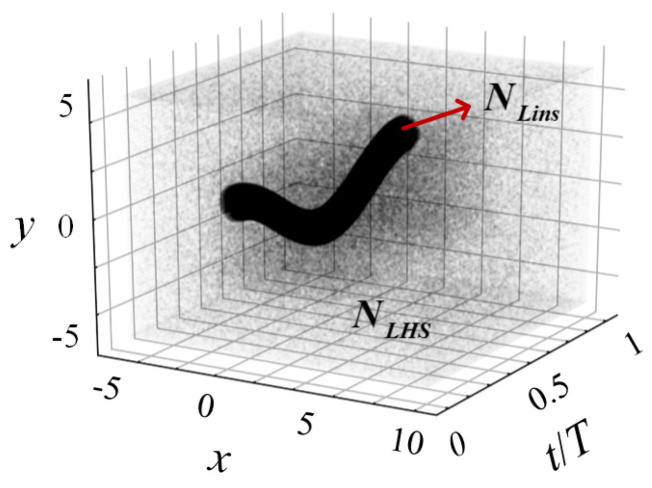
Illustration of the collocation points distribution adopted by the HCDD-PINN model. *N*_LHS_ represents the collocation points for the entire spatial–temporal space. *N*_Lins_ represents the instant collocation points around the flapping wing within a cycle shape.

The entire spatial–temporal space is filled with all the collocation points, and each point has certain spatial–temporal coordinates. These collocation points are generated and inputted into the DNN for training and to match the corresponding output variables. Similar to traditional CFD simulation, the training process requires a certain minimum number of collocation points to ensure the accuracy of PINN. The total collocation points $\;N_{collo}$ used for PINN can be calculated with Equation (18), which are shown in Figure 3 as a representation. That is,

(18) $$N_{collo}=N_{LHS}+timestep\cdot N_{Lins}$$

where $\;N_{LHS}$ and $\;N_{Lins}$ represent global and local collocation points, respectively. $\;N_{LHS}$ are obtained by taking a random sample using the Latin hypercube sampling (LHS) method. They are distributed in the entire spatial–temporal space, and are refined in the region of 7*c* × 5*c* (adjacent to the flapping wing) to better capture the details of the flow. $N_{Lins}$ are generated by the linspace method [14] within a cycle shape embracing the flapping wing. They only distributed in the local spatial space at a certain instant, to precisely illustrate the shape and the movements of the flapping wing. For the whole stroke cycle, they are sampled with uniform interval time, i.e., $N_{Lins}$ is generated for all the timestep. It is claimed that such a method for collocation-points generation can effectively improve the accuracy of PINN prediction for the current problem with a large body motion and unsteady features, which will be discussed later.

**Figure 4:**
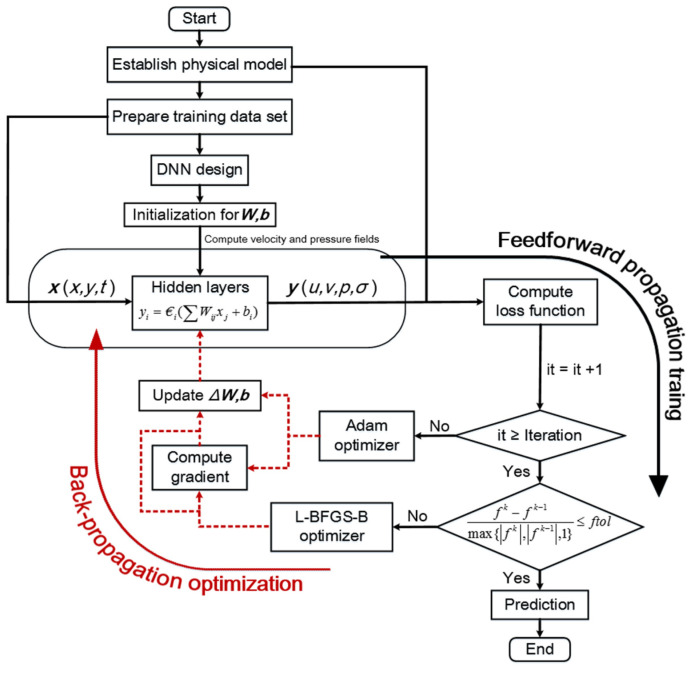
Flow chart for the HCDD-PINN training process.

A flow chat for the whole method can be viewed in Figure 4. The steps of the NN training process can be described as follows:

Step1: establish the physical model of flapping wing, including the governing equations, and boundary conditions, and prepare the training data for the coarser CFD results, including the initial condition and coarse internal data;
Step2: design the suitable deep neural network, including the NN structure, function, learning rate, iteration, and so on; and create the corresponding loss function according to the physical model;
Step3: train the DNN using the coarser internal data, and establish the map relationship between the input and output layers; use Adam and L-BFGS-B optimizers to update the weight and bias of DNN and reduce the error of the loss function; end training when the converged conditions are satisfied;
Step4: save the trained model, residual histories, and predicted flow field information.

## 3. Problem Setup and Numerical Results

### 3.1. Problem Description

In the current study, the flapping kinematics, *h_m_*, *α_m_*, and *f* in Equations (3) and (4), are fixed at 0.2*c*, π/4, and 0.25, respectively. *c* is the chord length of the wing, and the Reynolds number *Re* is based on the chord of the flapping wing; a free-stream velocity and a fluid viscosity of 100 are adopted. 

**Figure 5:**
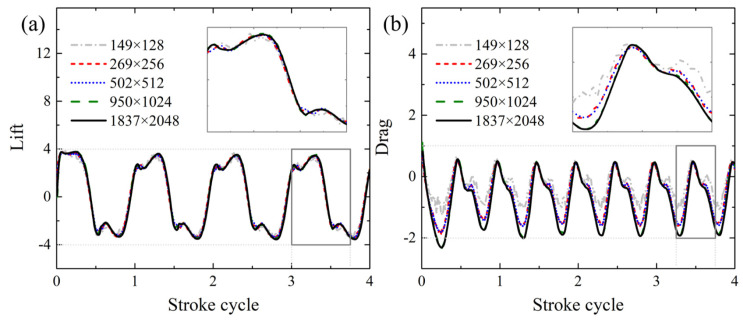
(**a**) The lift and (**b**) drag forces under different resolutions calculated by CFD solver.

A grid convergence study is performed by decreasing the minimum grid interval Δ*s* by a factor of 2, to ensure the CFD solution accuracy and prepare the PINN training data to validate the corresponding prediction performance of the proposed HCDD-PINN model. The corresponding lift and drag forces’ histories under different resolutions are compared in Figure 5. It can be seen that all the cases attain periodical state within three flapping cycles. Therefore, the CFD results from the fourth cycle are selected to be the data training and for comparison. For our PINN simulation, the fourth flapping cycle is trained and predicted. The reference field information at the beginning of the fourth cycle is used as the initial condition data (*t* = 0) to help with the PINN training, which introduces the initial condition loss $\varepsilon_{I}$ to the total loss. The high-resolution simulation (950 × 1024) is used as the ground truth and as a reference, and the three coarser-resolution simulations, i.e., 149 × 128, 269 × 256, and 502 × 512, are adopted as the coarse internal data, to improve the PINN’s capability and prediction accuracy, which introduces the coarse internal loss $\varepsilon_{int}$ to the total loss. The coarse internal data are scattered randomly at various spatial–temporal locations, which can be easily utilized in the DNN framework. 

### 3.2. Prediction Results

In this section, the effects of HCDD-PINN with different-resolution internal data are investigated, by comparing it with a PINN with no internal data. The three CFD results ((149 × 128), (269 × 256), and (502 × 512)) that are used as the coarse internal data are 51×, 14×, and 4× coarser resolution than the reference case (950 × 1024), and indicate ~259-fold, ~70-fold, and ~9-fold computational speedup, respectively. Three scalar state variables, i.e., velocity *u*, *v,* and pressure *p*, are saved in 25 field snapshots, and the number of snapshots used as internal data for PINN training is 25 for the three coarser cases. The fraction per snapshot is 0.0364, 0.01, and 0.00268, respectively, to ensure the same internal data are used for PINN. A case without internal data is also performed for comparison. To demonstrate the robustness of HCDD-PINN, the architecture and hyper-parameters are selected in a consistent fashion for all four of the cases. The architecture of the DNN used here is composed of eight hidden layers with 50 neurons per layer, to broadly balance the trade-off between the expressivity and trainability of the neural network. The weighting coefficients $\lambda_{bc/I}$ and $\lambda_{int}$ are set as one for both the initial and boundary conditions loss and the coarse internal loss. The total collocation point $N_{collo}$ is set to 3.1 × 10^5^ ${\;(N}_{LHS}=1{.6\;\times \;10}^{5}{,\;N}_{Lins}=1{.5\;\times \;10}^{3}$ , and timestep = 100). The learning rate and max batch size of Adam optimizer are set as 5 × 10^−4^ and 5 × 10^3^, respectively. The network construction, training, and prediction are all performed in Tensorflow. All of the training and predictions are performed on a high-performance computer cluster with 80 GB memory, twenty CPUs, and one NVIDIA Tesla V100 GPU. Note that a comprehensive parameter study and an analysis of the optimization of the DNN’s architecture will be conducted in the next section. 

The training and predicting performances on these four cases are summarized in Table 1, where the training loss is defined by Equation (11). The relative *l*_2_ error $rl_{2}e\_y$ of the output variables is defined as:

(19) $$rl_{2}e\_y=\frac{\sqrt{\sum_{i=1}^{N}{\left\|y_{PINN}^{i}-y_{CFD}^{i}\right\|}^{2}}}{\sqrt{\sum_{i=1}^{N}{\left\|y_{CFD}^{i}\right\|}^{2}}}$$

where ***y*** is the physical quantity of interest, including *u*, *v*, and *p*; *N* is the total number of the reference point. The predicting error is obtained by calculating the cycle-averaged relative *l*_2_ error $\bar{rl_{2}e\_y}$ ( $\bar{rl_{2}e\_y}=1/T\sum rl_{2}e\_y$ ) to evaluate the prediction performance. It can be seen from Table 1 that the PINN model without internal data inaccurately predicted the flow field past the flapping wing, despite the fact that the training loss had been minimized to less than 0.003. With the help of the limited coarse internal data, nearly 700 points per snapshot, the predicting error is largely reduced for all three of the other cases, at the cost of the increment in training cost and training loss. This is mainly caused by the introduction of $\varepsilon_{int}$ . The reference CFD simulation takes 1967 s to complete four stroke cycles under the same CPU cores as the PINN. The PINN training time increases ~5.8-fold (no internal), ~15.6-fold (149 × 128), ~9.4-fold (269 × 256), and ~6.8-fold (502 × 512), respectively. However, once the training process of HCDD-PINN model is finished, the flow information at arbitrary spatial–temporal coordinates can be rapidly predicted with reasonable accuracy. This will be suitable for wing-shape optimization, stroke-trajectory kinematics optimization, and aero-forces optimization. The advantages of HCDD-PINN will be more apparent when the situation is extended to the corresponding three-dimensional problems, which needs an excellent mesh quality to ensure the accuracy of the traditional CFD solvers. The 3D CFD simulations usually need to solve billions of degrees of freedom in the relevant spatial–temporal flow fields, leading to unfavorable trade-offs between accuracy and computational cost. 

**Figure 6:**
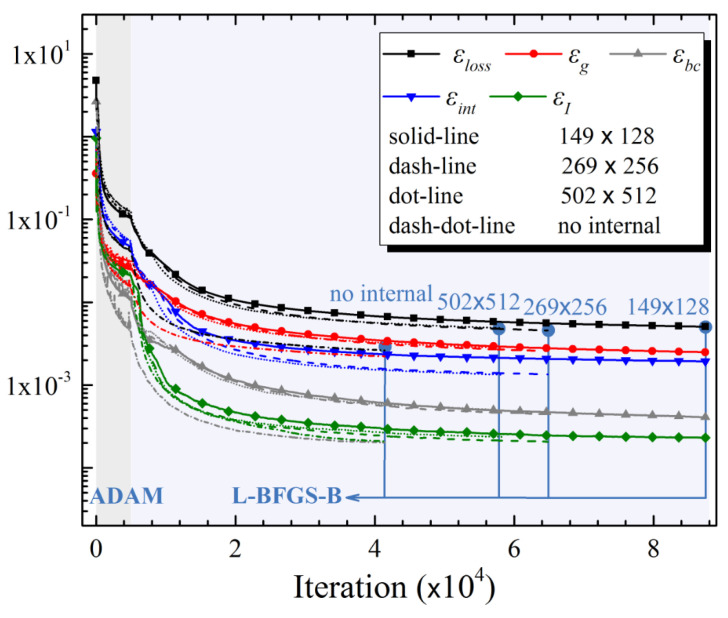
Required iterations during optimization of the total loss and other loss components in the training process of HCDD-PINN with different coarse internal data.

The weighted total residual for all cases is less than 0.0051. As expected, the coarser-resolution the internal data are, the more time and iterations the HCDD-PINN model needs to train. It is worth mentioning that the HCDD-PINN with 269 × 256 resolution internal data shows the best predicting performance compared to the other internal data resolutions, which results from the longer training time of the relatively accurate data with relatively few points. It can reconstruct the velocity field and the pressure field with relative accuracy. More details about the residual loss convergence histories, including the total loss $\;\varepsilon_{loss}$ , the governing equations loss $\;\varepsilon_{g}$ , the boundary condition loss $\;\varepsilon_{bc}$ , the initial condition loss $\;\varepsilon_{I}$ , and the coarse internal loss $\;\varepsilon_{int}$ , can be found in Figure 6. It requires nearly 60,000~90,000 iterations to reach a properly trained HCDD-PINN model that is deemed ready to be used for predictions. The sudden drop in the loss histories is caused by switching from the ADAM optimizer to the L-BFGS-B optimizer.

**Figure 7:**
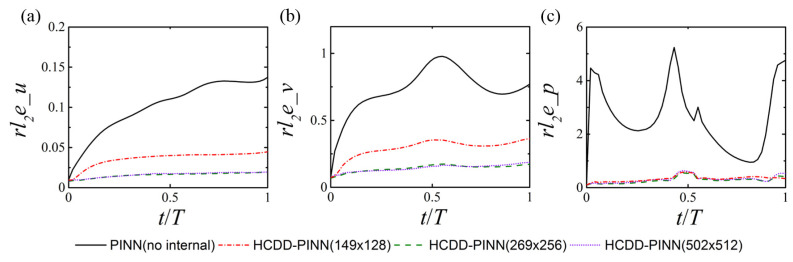
Relative l2 errors between the HCDD-PINN model predictions and the CFD reference for velocities (**a**) *u*, (**b**) *v*, and pressure (**c**) *p* fields. The results of the PINN model with no internal data are given for comparison.

After training is complete, the predictions of the velocity and pressure fields can be obtained almost instantaneously. Nearly one hundred thousand points scattered in space are used for prediction at a certain instant, and 51 time snapshots are saved for the entire flapping cycle, including the initial and final time instants. The corresponding prediction cost for the four cases can be found in Table 1. The relative *l*_2_ errors of velocity and pressure fields, between the predictions of the HCDD-PINN model with different coarse internal data resolutions and the exact values of the corresponding CFD reference, are calculated for one cycle, as illustrated in Figure 7. The results of the PINN model with no internal data are given for comparison. It is clear that the proposed framework is capable of constructing the entire velocity and pressure fields, and has significantly improved the predicting accuracy compared to the original PINN model. The HCDD-PINN with very coarse internal data (149 × 128) still has a good prediction, which validates that the proposed method possesses a high degree of robustness.

**Figure 8:**
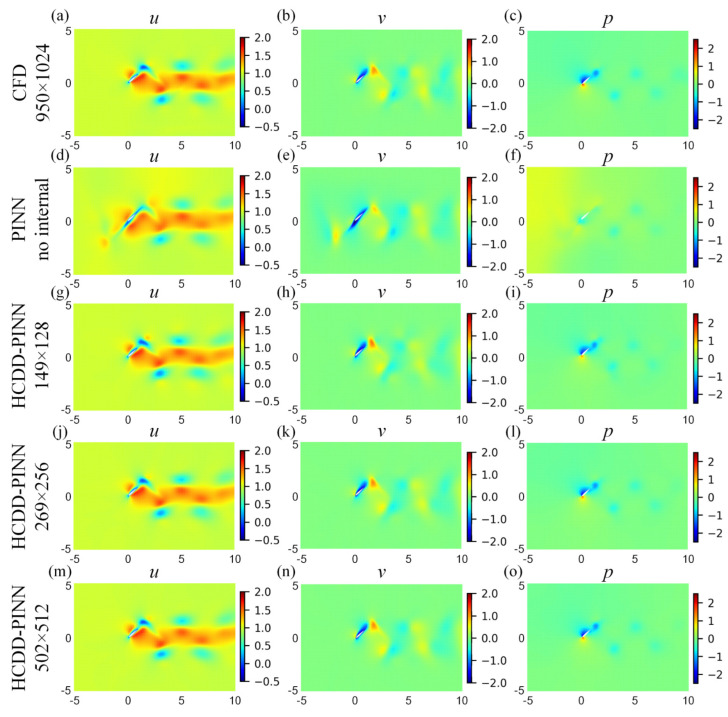
Cloud pictures for the predicted velocity (*u*-left column, *v*-middle column) and pressure (*p*-right column) fields of the HCDD-PINN model, with respect to different resolutions of coarse internal data at *t*/*T* = 0.18: (**a**–**c**) CFD reference with 950 × 1024; (**d**–**f**) PINN with no internal; (**g**–**i**) HCDD-PINN with 149 × 128; (**j**–**l**) HCDD-PINN with 269 × 256; (**m**–**o**) HCDD-PINN with 502 × 512.

**Figure 9:**
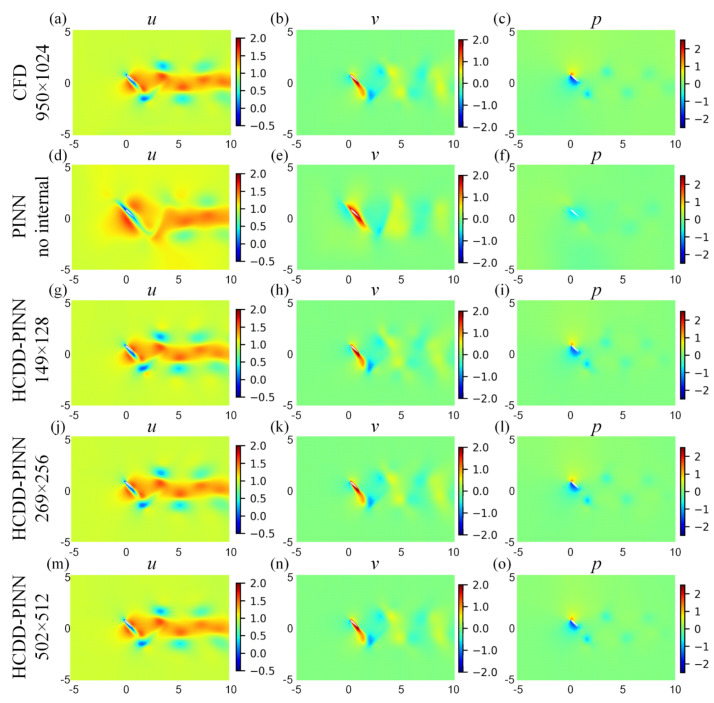
Cloud pictures for the predicted velocity (*u*-left column, *v*-middle column) and pressure (*p*-right column) fields of the HCDD-PINN model, with respect to different resolutions of coarse internal data at *t*/*T* = 0.78035: (**a**–**c**) CFD reference with 950 × 1024; (**d**–**f**) PINN with no internal; (**g**–**i**) HCDD-PINN with 149 × 128; (**j**–**l**) HCDD-PINN with 269 × 256; (**m**–**o**) HCDD-PINN with 502 × 512.

For a more in-depth investigation, cloud pictures for the predicted velocity (*u*, *v*) and pressure (*p)* fields of the HCDD-PINN model with different resolutions of coarse internal data are drawn at *t*/*T* = 0.18 and *t*/*T* = 0.78035, as shown in Figure 8 and Figure 9, respectively. They are compared with cases of no internal data and the ground truth reference. It can be seen that, compared to the PINN with no internal case, the flow fields predicted by the HCDD-PINN closely follow the governing partial differential equations laws and the enforced initial and boundary conditions at any instant, and the flows in the areas adjacent to the flapping wing and the wake can be precisely captured by the proposed framework.

**Figure 10:**
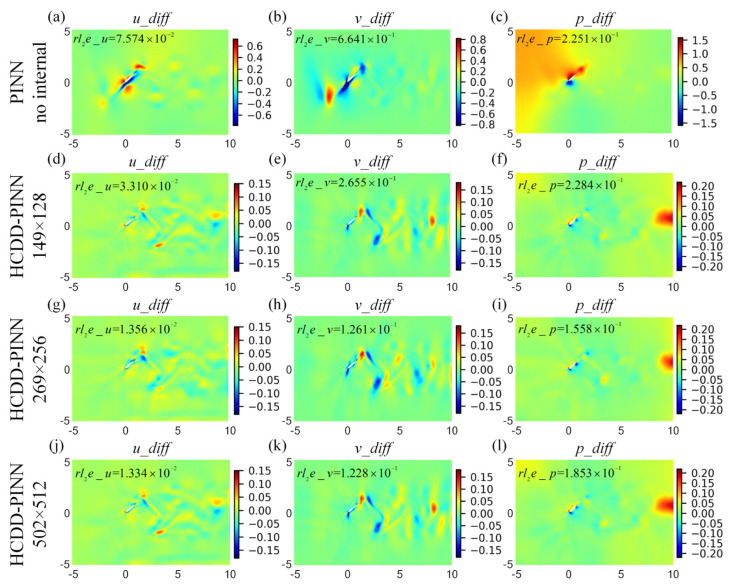
Absolute error cloud pictures for the velocity (*u_diff*-left column, *v_diff*-middle column) and pressure (*p_diff*-right column) flow fields between the CFD and HCDD-PINN, with respect to different coarse internal data at *t*/*T* = 0.18: (**a**–**c**) PINN with no internal; (**d**–**f**) HCDD-PINN with 149 × 128; (**g**–**i**) HCDD-PINN with 269 × 256; (**j**–**l**) HCDD-PINN with 502 × 512.

**Figure 11:**
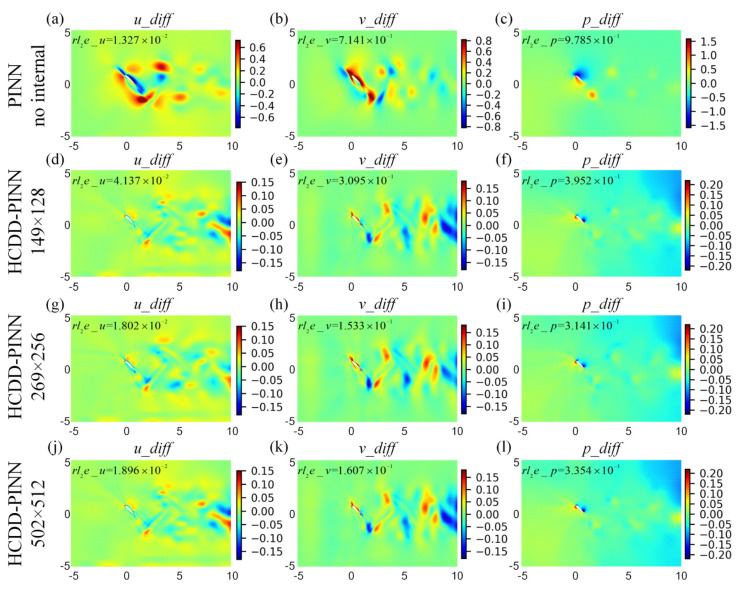
Absolute error cloud pictures for the velocity (*u_diff*-left column, *v_diff*-middle column) and pressure (*p_diff*-right column) flow fields between the CFD and HCDD-PINN, with respect to different coarse internal data at *t*/*T* = 0.78035: (**a**–**c**) PINN with no internal; (**d**–**f**) HCDD-PINN with 149 × 128; (**g**–**i**) HCDD-PINN with 269 × 256; (**j**–**l**) HCDD-PINN with 502 × 512.

To distinguish the differences of flow fields between the prediction and the ground truth, we define the absolute error as $y_{diff}=y_{PINN}-y_{CFD}$ . The corresponding absolute error cloud pictures for the *u*, *v* velocities and *p* pressure fields are drawn at *t*/*T* = 0.18 and *t*/*T* = 0.78035, as shown in Figure 10 and Figure 11, respectively. The positive absolute error, illustrated by the red color, means that the predicted values are larger than the extracted ones, while the blue negative error represents that the predicted values are smaller than the extracted ones. The corresponding relative *l*_2_ errors of *u_diff*, *v_diff*, and *p_diff*, defined by Equation (20), are given in the upper left corner of the respective picture for the whole domain. It can be seen that the HCDD-PINN models with different resolutions of coarse internal data are able to produce a good prediction performance compared to the original PINN model with no data to help. The relatively large absolute error occurs near the flapping wing and some specific areas, while the field information for the most parts of the domain is predicted with sufficient accuracy. The prediction of the velocity field is more accurate than that for the pressure field for all cases. The relatively large difference in magnitude between the exact and the predicted pressure fields may be attributed to the very nature of incompressible Navier–Stokes equations, since the pressure field is only identifiable up to a constant. 

**Figure 12:**
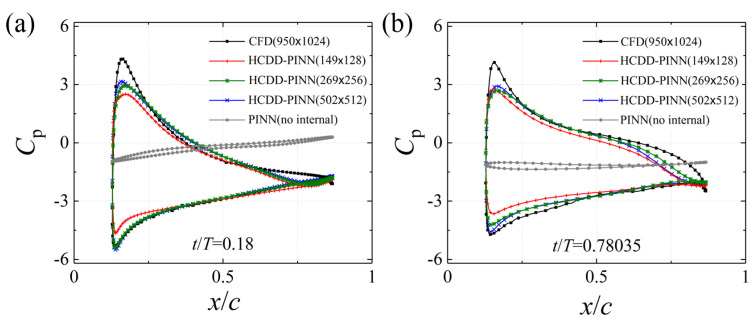
The distribution of the pressure coefficient *C*_p_ for the flapping wing predicted by the HCDD-PINN with respect to different coarse internal data at (**a**) *t*/*T* = 0.18 and (**b**) *t*/*T* = 0.78035. The results of the model with no internal data are given for comparison.

Comparisons of the distribution pressure coefficient *C*_p_ in the flapping wing, between the CFD reference values (black line) and PINN predicted values with no internal data (grey line), 149 × 128 (red line), 269 × 256 (green line), and 502 × 512 (blue line) coarse internal data, at *t*/*T* = 0.18 and *t*/*T* = 0.78035, are illustrated in Figure 12a and 12b, respectively. The pressure coefficient *C*_p_ is obtained by dividing $0.5\rho U_{ref}^{2}c$ by the pressure force. Despite the relatively large difference between the predicted and exact pressure fields, the distribution of the pressure coefficient *C*_p_ for the flapping wing appears to be accurately predicted by the proposed HCDD-PINN with limited coarse scattered data.

## 4. Discussions

Firstly, in this section, an optimal parameter-search study is conducted to find the best DNN parameters for the flapping wing problem. Secondly, the effects of coarse internal data on the training and prediction performances, with respect to different snapshots and fractions of internal data, are investigated by adopting the best combination of these parameters. Based on the above analysis, the coarse results obtained with the 269 × 256 grid resolution are selected as the internal data source for the HCDD-PINN model in this section, as it gives the best predicting performance.

### 4.1. Optimal Parameters Search

The training and predicting performances of DNNs are significantly affected by the architecture, hyper-parameters, and the number of collocation points, which are optimized by a coarse grid search. A summary of the training and predicting performances for all the test experiments are presented in Table 2. The baseline parameters were selected to be snapshot = 25, fraction = 0.01, $N_{LHS}=1{.6\;\times \;10}^{5},\;N_{Lins}=1{.5\;\times \;10}^{3},$ timestep = 100, layer = 8, neurons per layer = 50, $\lambda_{bc/I}\;$ = 1, $\lambda_{int\;}$ = 1, learning rate = 5 × 10^−4^, and iteration = 5 × 10^3^. All these test parameters are varied individually by fixing the other parameters. 

It can be seen from Table 2 that, as $N_{LHS}$ increases, the more training time is needed, but the training loss and the predicting errors are decreased. $N_{LHS}$ of 2.4 $\times {\;10}^{5}$ is selected by weighting the computational cost and the prediction accuracy. It can be seen that the introduction of $N_{Lins}$ can significantly improve the HCDD-PINN’s capability of predicting the flow past the flapping wing. However, the improvement tends to slow down while using larger points of $N_{Lins}$ . $N_{Lins}\;$ of 500 and timestep of 50 are selected in conformity with the smallest-collocation-points principle, which contributes to the total $N_{collo}$ of 2.65 $\times {\;10}^{5}$ . 

The empirical findings indicate that deeper and wider networks are usually more expressive (i.e., they can capture a larger class of functions, but are often costlier to train, which represents a feed-forward evaluation as the neural network takes more time and the optimizer requires more iterations to converge). A large number of layers prevents a model from being generalized, while a small number of layers is insufficient to represent the system. Therefore, an architecture of DNN with the minimum number of layers and neurons and the desired performance is preferable. Among all cases considered here, the best structure of the HCDD-PINN includes six hidden layers with 100 neurons per layer. 

With the loss weighting coefficients increasing, the corresponding loss terms decrease. Considering that the information in the internal data is inaccurate, it is unnecessary to reduce the internal loss to a very low value. Therefore, the combination of a $\lambda_{bc/I}$ of three and a $\lambda_{int}$ of two are suitable to balance the weights of different items in the total residual. Additionally, the learning rate and the max number of iterations in the ADAM optimizer are 5 $\times {\;10}^{-5}$ and 1 $\times {\;10}^{4}$ , respectively, resulting in the better prediction performance of our proposed framework.

In summary, throughout the coarse grid search study, with the snapshot and fraction of coarse internal data fixed as 25 and 0.01, the best combination of the other parameters for DNN are $N_{LHS}=2{.4\;\times \;10}^{5},\;N_{Lins}=500,$ timestep = 50, layer = 6, neurons per layer = 100, $\lambda_{bc/I}\;$ = 3, $\lambda_{int}\;$ = 2, learning rate = 5 × 10^−5^, and iteration = 5 × 10^4^. These sets of parameters will be adopted when investigating the effects of the fraction and snapshot of coarse internal data in the next section.

### 4.2. Effect of the Fraction of Coarse Internal Data

A train-and-test data split method is used for preparing the coarse internal data of the HCDD-PINN. The fraction parameter illustrates the percentage of the training data in the source CFD data, and the test data are ignored in the training process. The effect of the fraction of coarse internal data on the training and predicting performances of our proposed HCDD-PINN model is investigated by fixing the snapshot number of the coarse internal data to 25. The source of the coarse internal data is a 269 × 256 coarse grid CFD simulation, and five values of the fraction are considered for each snapshot, i.e., 0.1, 0.05, 0.01, 0.005, and 0.001. That is to say, the total internal points at each snapshot are nearly 6900, 3450, 690, 345, and 69, respectively. 

**Figure 13:**
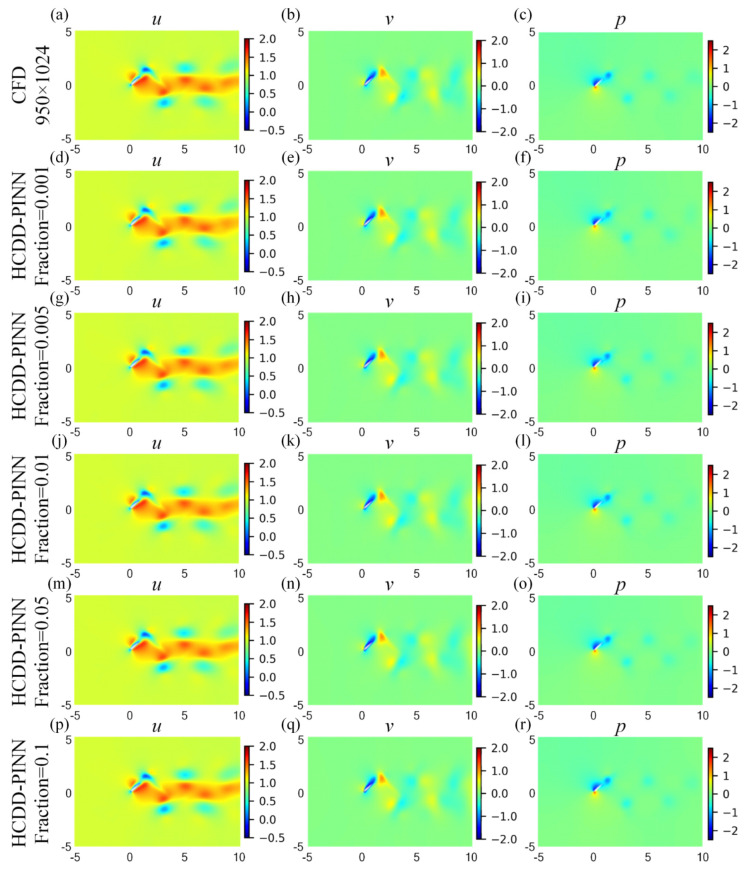
Cloud pictures for the predicted velocity (*u*-left column, *v*-middle column) and pressure (*p*-right column) fields of the HCDD-PINN model, with respect to different fraction of coarse internal data at *t*/*T* = 0.18: (**d**–**f**) fraction = 0.001; (**g**–**i**) fraction = 0.005; (**j**–**l**) fraction = 0.01; (**m**–**o**) fraction = 0.05; (**p**–**r**) fraction = 0.1. Results of (**a**–**c**) CFD reference with 950 × 1024 are given for comparison.

The overall training and predicting results of HCDD-PINN, with different fractions of coarse internal data, are summarized in Table 3. Compared to the case with the old DNN parameters, the HCDD-PINN with optimal parameters has a better predicting performance with an increment in the cost of training time. The weighted total residuals throughout this section are minimized to be less than 0.0025. The overall effect of the fraction is relatively small. As the fraction increases, the predicting errors of pressure can be effectively decreased. Except for the pressure field, the training and predicting performances of the HCDD-PINN hardly benefit from the large fraction of the coarse internal data. On the contrary, the HCDD-PINN can predict the flow fields of the flapping wing with relative accuracy with a limited dataset, as long as the internal points at each snapshot are larger than a hundred. It can also be seen in Figure 13, which exhibits cloud pictures for the predicted and exact velocity and pressure at *t*/*T* = 0.18, with respect to different fractions, that the proposed HCDD-PINN model is robust for accurately reconstructing the velocity and pressure flow fields when the fraction of coarse internal data varies. 

**Figure 14:**
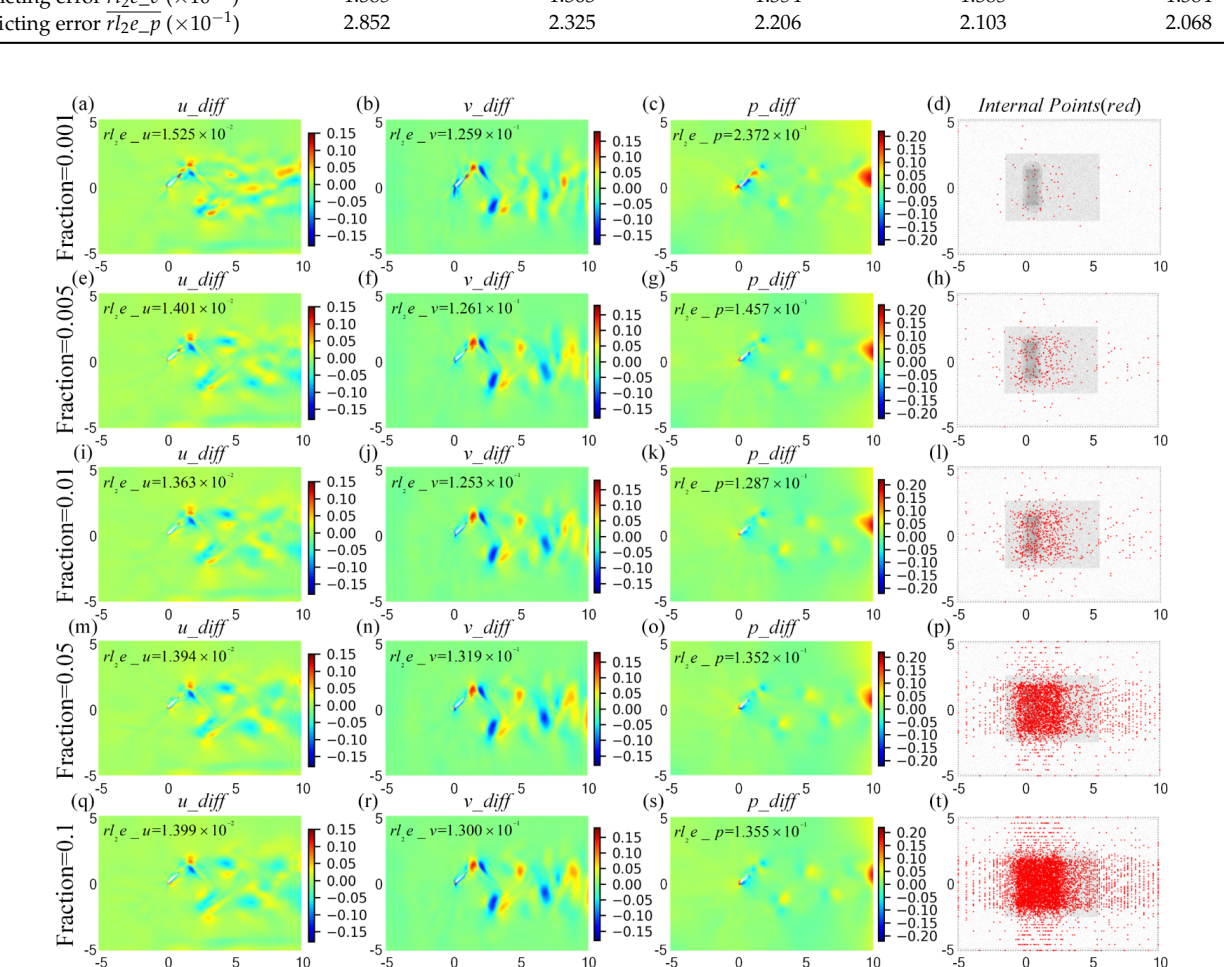
Absolute error cloud pictures for the velocity (*u_diff*-left column, *v_diff*-middle column) and pressure (*p_diff*-right column) flow fields between the CFD and HCDD-PINN, with respect to different fraction of coarse internal data at *t*/*T* = 0.18: (**a**–**c**) fraction = 0.001; (**e**–**g**) fraction = 0.005; (**i**–**k**) fraction = 0.01; (**m**–**o**) fraction = 0.05; (**q**–**s**) fraction = 0.1. The corresponding relative *l*_2_ errors are given below the picture. The internal data used for different fractions are illustrated by red points at the corresponding position of the last column (**d**,**h**,**l**,**p**,**t**).

The differences in *u*, *v* velocities, and *p* pressure fields are compared among the predictions of the HCDD-PINN with different fractions of coarse internal data and the exact CFD reference, which are given as *t*/*T* = 0.18 in Figure 14. The corresponding relative *l*_2_ errors are illustrated below each respective picture. It can be seen that the absolute errors of *u* and *v* rarely change, with respect to the different fractions of the coarse internal data. The absolute error of *p* increases to a certain extent as the fraction decreases from 0.1 to 0.001. Considering that the internal points are reduced by nearly 100 times, such adverse effects are still deemed to be acceptable by weighting the data-driven amount and predicting accuracy. The predicting capability of the proposed HCDD-PINN model is stable enough only if an appropriate snapshot number of coarse internal data is selected, no matter how much the fraction changes.

### 4.3. Effect of the Snapshot Number of Coarse Internal Data

To investigate the effects of the snapshot number of coarse internal data, seven values are considered for the snapshots of coarse internal data, i.e., twenty-fie, thirteen, nine, seven, five, four, and three. In particular, the snapshot = 0 represents the original PINN model without internal data. The fraction of coarse internal data is adopted as 0.005 to ensure the internal data are in the order of hundreds. The source CFD data are saved in 25 field snapshots, and the timestep per cycle of the HCDD-PINN is 50. Therefore, nearly every two, four, six, eight, twelve, sixteen, and twenty-four timestep intervals, the corresponding coarse internal data at a certain instant are introduced into the HCDD-PINN for training, respectively. The summaries of the training and predicting results of HCDD-PINN with different snapshots of coarse internal data are given in Table 3. The training loss is observed to increase, while a decrease is detected for the predicting errors and the training cost, as the snapshot of the coarse internal data increases. When all 25 of the coarse internal data snapshots are used for the model training, it would take the shortest training time to minimize the loss function to the lowest value, and perform best in reconstructing the flow fields as expected. The flow fields predicted by the original PINN model without internal data include large errors compared to the ground truth, while the predicting errors of HCDD-PINN sharply decrease with the inclusion of coarse internal data. When gradually adding the snapshot of coarse internal data to more than four, the predicting errors are reduced to an acceptable number, and the predicting performances remain stable as the snapshot increases. 

**Figure 15:**
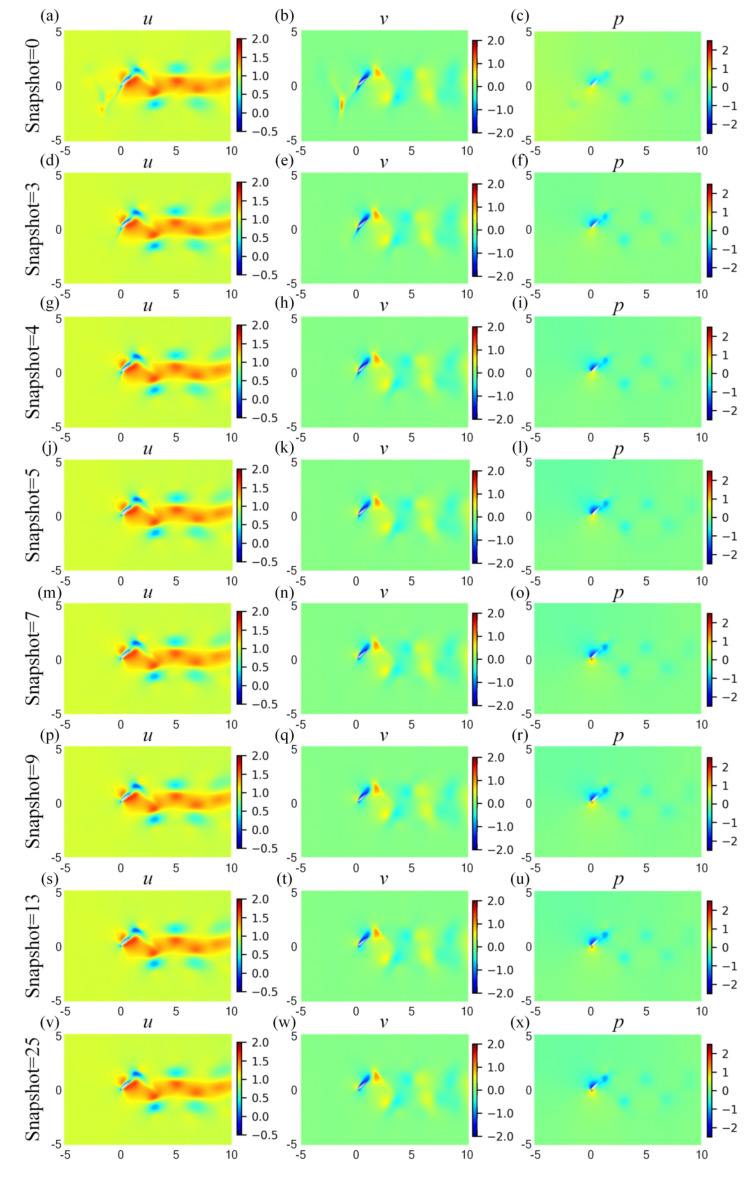
Cloud pictures for the predicted velocity (*u*-left column, *v*-middle column) and pressure (*p*-right column) fields of the HCDD-PINN model, with respect to different snapshot of coarse internal data at *t*/*T* = 0.18: (**a**–**c**) snapshot = 0; (**d**–**f**) snapshot = 3; (**g**–**i**) snapshot = 4; (**j**–**l**) snapshot = 5; (**m**–**o**) snapshot = 7; (**p**–**r**) snapshot = 9; (**s**–**u**) snapshot = 13; (**v**–**x**) snapshot = 25.

**Figure 16:**
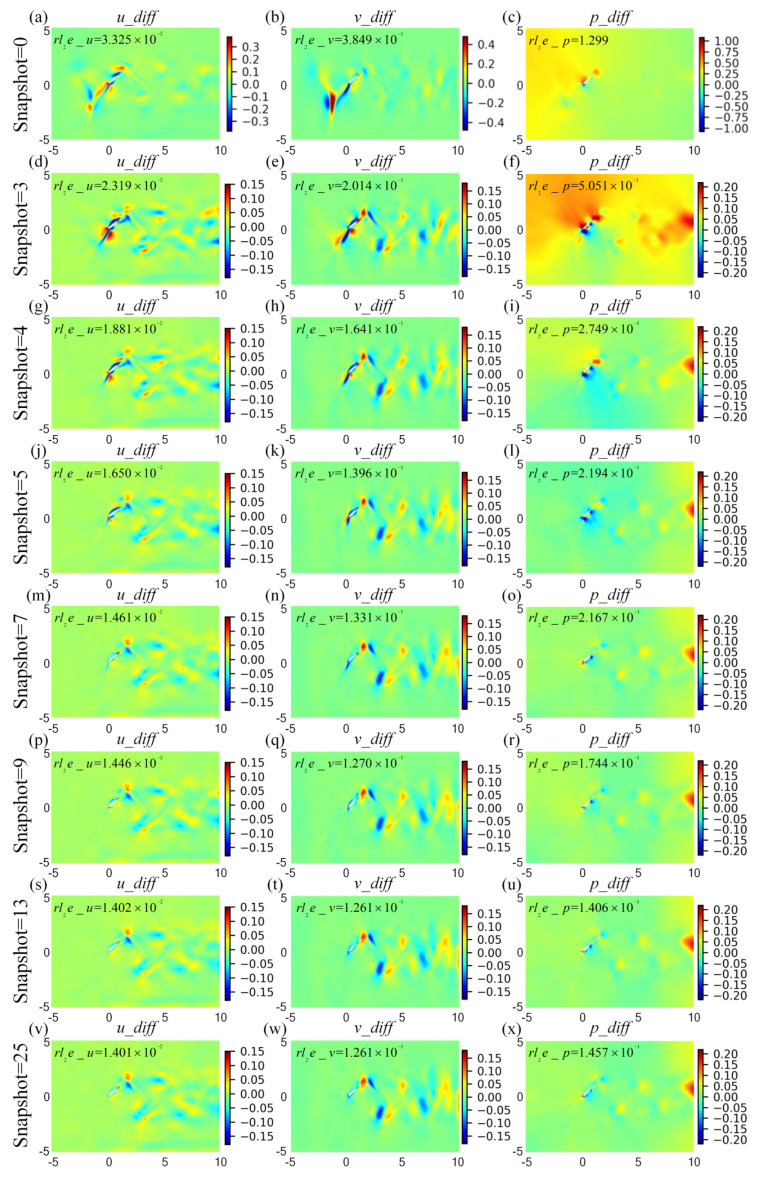
Absolute error cloud pictures for the velocity (*u_diff*-left column, *v_diff*-middle column) and pressure (*p_diff*-right column) flow fields between the CFD and HCDD-PINN, with respect to different snapshot of coarse internal data at *t*/*T* = 0.18: (**a**–**c**) snapshot = 0; (**d**–**f**) snapshot = 3; (**g**–**i**) snapshot = 4; (**j**–**l**) snapshot = 5; (**m**–**o**) snapshot = 7; (**p**–**r**) snapshot = 9; (**s**–**u**) snapshot = 13; (**v**–**x**) snapshot = 25. The corresponding relative *l*_2_ errors are given below the picture.

The cloud pictures for the velocity and pressure fields at *t*/*T* = 0.18, predicted by HCDD-PINN with respect to different snapshots of coarse internal data, are summarized in Figure 15. It can be seen that the proposed HCDD-PINN model can accurately reconstruct the velocity and pressure flow fields by utilizing the coarse internal data. The flow information can be captured with sufficient accuracy, especially near the leading edge of the flapping wing, as long as the snapshot of coarse internal data is increased up to five. It can be better distinguished by drawing the corresponding cloud pictures of the differences between the predicted and exact velocity and pressure fields at this moment, as shown in Figure 16. It can clearly be concluded that the absolute errors between the HCDD-PINN and CFD decrease significantly when the snapshot of coarse internal data changes from 0 to 25.

## 5. Conclusions

Traditional deep learning requires a large amount of training data to solve the flow problems, which is usually a very time-consuming process. According to the known partial differential equations, physics-constrained deep learning has the potential to create faster and more accurate solutions for fluid flow over an arbitrary domain of interest. However, the existing PINN model may generate unreasonable or unrealistic predictions for specific problems due to the innate lack of understanding in the complexities of neural network. In the current study, taking the flow past the flapping wing as a specific example, a hybrid coarse-data-driven physics-informed neural network (HCDD-PINN) model is proposed for solving such highly unsteady problems with large body motions. The sources of internal data are coarser in magnitude than is required by the traditional high-resolution CFD. The training and predicting performances of HCDD-PINN with different resolutions of internal data are analyzed, by comparing it to the original PINN model without internal data. Additionally, the effects of the snapshot and fraction of the coarse internal data on the HCDD-PINN performances are investigated, based on the best-parameters combination of DNN obtained from an optimal parameter-search study.

By introducing the coarse internal data, the proposed HCDD-PINN model has sufficient accuracy, and performs reasonable and realistic predictions of the flow past the flapping wing. The results are compared to the ground-truth reference, which is obtained by an immersed-boundary method-based solver. In general, the velocity and pressure fields can be precisely predicted by HCDD-PINN with different grid resolutions for the coarse internal data. The absolute errors of velocity are lower than that of pressure *p*. The errors in predicting pressure can be effectively decreased by appropriately increasing the snapshot and fraction of the coarse internal data. The proposed HCDD-PINN framework is considered to have sufficient stability and accuracy for solving specific problems with unsteady features and large body motions. This method offers an alternative way to solve arbitrarily complex fluid dynamic problems. Its advantages in accuracy, stability, and efficiency are likely to be even more apparent for the corresponding optimization and three dimensional researches, since they are notoriously time consuming to solve with the standard CFD methods; this issue awaits our future work. 

## Figures and Tables

**Table 1:** Training and predicting performance for HCDD-PINN with different coarse internal data.

	No Internal	Coarse Internal Data Obtained from Different Resolution		
		149 **×** 128	269 **×** 256	502 **×** 512
Training cost	11,412 s	30,643 s	18,465 s	13,427 s
Prediction cost	298 s	314 s	345 s	311 s
Training loss $\varepsilon_{loss}$	2.64 × 10^−3^	5.08 × 10^−3^	4.59 × 10^−3^	4.90 × 10^−3^
Predicting error $\bar{rl_{2}e\_u}\;$	1.01 × 10^−1^	3.63 × 10^−2^	1.56 × 10^−2^	1.61 × 10^−2^
Predicting error $\bar{rl_{2}e\_v}\;$	7.25 × 10^−1^	2.92 × 10^−1^	1.43 × 10^−1^	1.44 × 10^−1^
Predicting error $\bar{rl_{2}e\_p}\;$	2.59	3.32 × 10^−1^	2.75 × 10^−1^	3.12 × 10^−1^

**Table 2:** Training and predicting performance of HCDD-PINN for optimal parameters search.

Collocation points $N_{collo}=N_{LHS}+timestep\cdot N_{Lins}$																																													
$N_{LHS}$ ( ${\times 10}^{5}$ )	1.6					2.4					3.2					1.6					1.6					1.6					1.6					1.6					1.6				
$N_{LinS}$	1500					1500					1500					0					500					2500					1500					1500					1500				
timestep	100					100					100					100					100					100					50					150					200				
Training cost ( ${\times 10}^{4}$ s)	1.847					2.651					3.186					1.798					1.896					2.805					1.018					2.794					3.070				
Training loss $\varepsilon_{loss}\;\left(\times 10^{-3}\right)$	4.592					3.649					3.356					80.24					3.155					4.114					4.077					4.464					5.068				
Predicting error $\bar{rl_{2}e\_u}\;$ ( ${\times 10}^{-2}$ )	1.564					1.540					1.532					3.256					1.528					1.544					1.507					1.581					1.607				
Predicting error $\bar{rl_{2}e\_v}\;$ ( ${\times 10}^{-1}$ )	1.433					1.411					1.390					6.326					1.411					1.407					1.412					1.441					1.450				
Predicting error $\bar{rl_{2}e\_p}\;$ ( ${\times 10}^{-1}$ )	2.753					2.520					2.468					4.821					2.596					2.557					2.657					2.614					2.757				
Architecture of DNN																																													
Layer	6															8															10														
Neurons (per layer)	50					100					150					50					100					150					50					100					150				
Training cost ( ${\times 10}^{4}$ s)	2.273					2.944					2.944					1.847					2.235					3.403					1.640					2.835					5.082				
Training loss $\varepsilon_{loss}\;\left(\times 10^{-3}\right)$	6.136					2.686					2.720					4.592					3.344					2.997					4.773					3.544					2.677				
Predicting error $\bar{rl_{2}e\_u}\;$ ( ${\times 10}^{-2}$ )	1.630					1.537					1.511					1.564					1.551					1.546					1.567					1.562					1.551				
Predicting error $\bar{rl_{2}e\_v}\;$ ( ${\times 10}^{-1}$ )	1.461					1.383					1.385					1.433					1.438					1.437					1.474					1.436					1.415				
Predicting error $\bar{rl_{2}e\_p}\;$ ( ${\times 10}^{-1}$ )	2.794					2.333					2.377					2.753					2.558					2.511					2.743					2.698					2.571				
Loss weighting coefficients																																													
$\lambda_{bc/I}$	1									2									3									1									1								
$\lambda_{int}$	1									1									1									2									3								
Training cost ( ${\times 10}^{4}$ s)	1.847									1.715									1.747									1.430									1.620								
Training loss $\varepsilon_{loss}\;\left(\times 10^{-3}\right)$	4.592									4.820									4.815									5.600									6.269								
$\varepsilon_{g}$ ( ${\times 10}^{-3}$ )	2.585									2.781									2.813									3.037									3.228								
${\varepsilon_{bc}(\times 10}^{-4}$ )	4.466									1.990									1.206									5.602									6.476								
$\varepsilon_{I}$ ( ${\times 10}^{-4}$ )	2.075									1.115									0.827									2.197									2.837								
$\varepsilon_{int}$ ( ${\times 10}^{-3}$ )	1.353									1.418									1.392									0.891									0.703								
Predicting error $\bar{rl_{2}e\_u}$ ( ${\times 10}^{-2}$ )	1.564									1.606									1.539									1.568									1.592								
Predicting error $\bar{rl_{2}e\_v}$ ( ${\times 10}^{-1}$ )	1.433									1.419									1.427									1.412									1.430								
Predicting error $\bar{rl_{2}e\_p}\;$ ( ${\times 10}^{-1}$ )	2.753									2.774									2.659									2.420									2.283								
Adam optimizer																																													
Learning rate ( ${\times 10}^{-4}$ )	5									1									0.5									5									5								
Iteration ( ${\times 10}^{3}$ )	5									5									5									10									15								
Training cost ( ${\times 10}^{4}$ s)	1.847									1.585									1.693									1.934									2.185								
Training loss $\varepsilon_{loss}\;\left(\times 10^{-3}\right)$	4.592									4.801									4.500									4.167									4.392								
Predicting error $\bar{rl_{2}e\_u}\;$ ( ${\times 10}^{-2}$ )	1.564									1.562									1.543									1.544									1.590								
Predicting error $\bar{rl_{2}e\_v}\;$ ( ${\times 10}^{-1}$ )	1.433									1.432									1.424									1.424									1.421								
Predicting error $\bar{rl_{2}e\_p}\;$ ( ${\times 10}^{-1}$ )	2.753									2.711									2.620									2.610									2.633								

**Table 3:** Training and predicting performance of HCDD-PINN with respect to different snapshots and fraction of the coarse internal data.

Coarse internal data (269 × 256)																																								
	Snapshot (fraction = 0.005)																																							
	0					3					4					5					7					9					13					25				
Training cost (s)	35,694					30,926					28,742					28,495					28,010					27,624					23,496					22,712				
Training loss $\varepsilon_{loss}\;\left({\times 10}^{-3}\right)$	0.806					1.405					1.512					1.599					1.658					1.683					1.902					2.079				
Predicting error $\bar{rl_{2}e\_u}\;$ ( ${\times 10}^{-2}$ )	5.311					2.064					1.751					1.682					1.584					1.569					1.554					1.537				
Predicting error $\bar{rl_{2}e\_v}\;$ ( ${\times 10}^{-1}$ )	4.193					1.770					1.568					1.464					1.415					1.396					1.380					1.363				
Predicting error $\bar{rl_{2}e\_p}\;$ ( ${\times 10}^{-1}$ )	1.435					4.857					4.108					2.879					2.749					2.597					2.375					2.325				
	Fraction (snapshot = 25)																																							
	0.001								0.005								0.01								0.05								0.1							
Training cost (s)	26,267								22,712								24,717								24,629								33,146							
Training loss $\varepsilon_{loss}$ $\left({\times 10}^{-3}\right)$	1.676								2.079								2.447								2.406								2.017							
Predicting error $\bar{rl_{2}e\_u}\;$ ( ${\times 10}^{-2}$ )	1.631								1.537								1.532								1.540								1.534							
Predicting error $\bar{rl_{2}e\_v}\;$ ( ${\times 10}^{-1}$ )	1.385								1.363								1.354								1.385								1.384							
Predicting error $\bar{rl_{2}e\_p}\;$ ( ${\times 10}^{-1}$ )	2.852								2.325								2.206								2.103								2.068							

## Data Availability

The author declares that there is no other available data.

## Nomenclature

*c*	chord length, m
$C_{P}$	pressure coefficient
*f*	stroke frequency, s^−1^
*h*_m_	plunge amplitude, m
mse_u	mean square error of *u*
mse_v	mean square error of *v*
mse_p	mean square error of *p*
$\bar{mse\_u}$	cycle-averaged mean square error of *u*
$\bar{mse\_v}$	cycle-averaged mean square error of *v*
$\bar{mse\_p}$	cycle-averaged mean square error of *p*
$N_{collo}$	total collocation points
$N_{LHS}$	collocation points for the entire spatial-temporal space
$N_{Lins}$	instant collocation points around the flapping wing
*Re*	Reynolds number
*T*	stroke period, s
*α*_m_	pitch amplitude, **°**
$\varepsilon_{bc}$	boundary condition loss
$\varepsilon_{g}$	governing equations loss
$\varepsilon_{int}$	coarse internal data loss
$\varepsilon_{I}$	initial condition loss
$\varepsilon_{loss}$	governing equations loss
$\lambda_{bc/I}$	weighting coefficient for initial and boundary condition losses
$\lambda_{int}$	weighting coefficient for coarse internal data loss
*σ*	Cauchy stress tensor
